# Insights into leprosy epidemiology from an isolated population located in the Brazilian Amazon

**DOI:** 10.1038/s41598-025-90399-0

**Published:** 2025-02-19

**Authors:** Ciane Cristina de Oliveira Mackert, Fernando Panissa Lázaro, Márcia Olandowski, Helena Regina Salomé D’Espindula, Andressa Mayra dos Santos, Priscila Verchai Uaska Sartori, Rafael Saraiva de Andrade Rodrigues, Geison Cambri, Marília Brasil Xavier, Erwin Schurr, Alexandre Alcaïs, Marcelo Távora Mira

**Affiliations:** 1 Graduate Program in Health Sciences, School of Medicine and Life Sciences, Pontifícia Universidade Católica do Paraná, Curitiba, PR Brazil; 2 Core for Tropical Medicine, Federal University of Pará, Belém, PA Brazil; 3 Infectious Diseases and Immunity in Global Health, McGill University Health Centre, Montreal, Canada; 4 Laboratory of Human Genetics of Infectious Diseases, Institut National de la Santé et de la Recherche Medicale and University Paris René Descartes, Necker Medical School, Paris, France; 5 Department of Clinical Analysis, Universidade Federal do Paraná, Curitiba, PR Brazil

**Keywords:** Leprosy, Population, Epidemiology, Innate susceptibility, Infectious diseases, Epidemiology

## Abstract

Leprosy, a chronic infectious disease caused mainly by *Mycobacterium leprae* (*M. leprae*), is still an important public health problem in countries such as Brazil and India. Here, we estimate key epidemiological parameters in the Prata Village, a unique, hyper-endemic, former leprosy colony isolated in the Brazilian Amazon. This is a population-based study in which the entire Prata Village population has been enrolled. Clinical, socioeconomic, and demographic data were obtained and validated by cross-checking using three independent information sources. Validated data was used for descriptive epidemiological analysis. From a total of 2,005 inhabitants by the time of the enrollment, 1,084 (56.2%) were born in the Village and, therefore, likely under lifelong exposure to leprosy cases. We observed differences between the sub-populations born and not born in the village in the cumulative prevalence of leprosy (5.9% vs. 22.9%, respectively) and the median age at diagnosis (15 years vs. 28 years, respectively). In contrast, there was no difference in the distribution of cases between males and females. Although extrapolating our findings to more open populations must be done carefully, we believe we used a unique population as a model to provide additional insights into the epidemiology of leprosy.

**Supplementary Information:**

The online version contains supplementary material available at 10.1038/s41598-025-90399-0.

## Introduction

Leprosy is a chronic infectious disease caused mainly by *Mycobacterium leprae* (*M. leprae*), an obligate and highly specialized intracellular bacteria that primarily infects skin macrophages and Schwann cells in the peripheral nervous system^1^. According to the Ridley-Jopling classification system, leprosy manifests clinically in a spectrum consisting of two polar—tuberculoid and lepromatous—and three intermediate borderline forms^2^. The latest data from the World Health Organization (WHO) indicates 182,815 newly reported cases of leprosy in 2023^3^, mainly concentrated in India and Brazil. The disease is effectively treated through multidrug therapy (MDT) following a WHO protocol considering the number of lesions and the bacillary load: paucibacillary patients (PB) present up to five skin lesions, and the absence of bacilli in bacilloscopy or histopathology, whereas multibacillary (MB) patients have a positive bacilloscopy and/or six or more lesions^4^.

The village of *Santo Antônio do Prata* (the Prata Village) was established in 1898 by Franciscan monks in the Amazon forest of the Northern Brazilian state of Pará. In 1923, the location was designated a compulsory isolation site for individuals diagnosed with leprosy from Brazil’s Northern and Northeastern states. Compulsory isolation was lifted in 1962^5^; however, due to the strong social stigma associated with the disease, the emigration of affected individuals and the immigration of non-affected families to the village has been limited over the decades. Consequently, the Prata Village remained socially and geographically isolated until recent years. The population of the village concentrates in an area of around 10 km^2^, and all residents share the same limited social infrastructure, such as a single church, two elementary schools, one social club, and a large central square serving as a communal gathering space for leisure activities such as soccer games and outdoor celebrations. Despite extensive efforts to reduce its incidence, leprosy remains highly prevalent and uniformly distributed throughout the community^6^. In 2010, a complex segregation analysis provided evidence of a significant major gene effect controlling susceptibility to leprosy within the Prata population, a finding compatible with the hypothesis that, given the history of the village, genetic risk factors for leprosy are enriched within this population^6^.

We propose that Prata village can serve as a compelling epidemiological model for leprosy, given that (i) the village is confined in a small, geographically isolated area, (ii) it is a leprosy hyperendemic cluster, (iii) the disease is distributed homogenously across the entire village, (iv) the population shares few social interaction spaces, consequently, (v) exposure to the disease is likely homogeneous and widespread among the community. Finally, (vi) the Prata population is enriched in genetic risk factors controlling leprosy susceptibility. Here, we present a population-based descriptive epidemiological study involving the entire population of the Prata Village.

## Methods

Enrollment of the entire population of the Prata Village was performed between 2006 and 2007; a detailed description of the enrollment strategy can be found in Lázaro F.P. et al. 2010^6^. In brief, every household in the village was visited by a research team member, and all adult members of resident families were interviewed; the parents or legal guardians provided information about individuals younger than 18 years old. Socio-economic data has been collected through a questionnaire. All individuals self-reported their present and previous leprosy status as affected or non-affected. All self-reported leprosy cases, active and historical, have been checked using three independent sources available at the village’s health unit: (i) the original medical records, (ii) copies of the compulsory notification forms, and (iii) a logbook containing registries of all cases for treatment follow-up. Self-reported cases confirmed at all three sources were validated as leprosy cases; clinical information was obtained from the medical records upon confirmation. The research was conducted in accordance with relevant guidelines and regulations, as outlined in the Helsinki Declaration (2008 revision). All subjects enrolled in the study agreed to participate and signed a written informed consent. The research project was approved by the Research Ethics Committee of the Pontifícia Universidade Católica do Paraná (51/07/CEP-PUCPR) and the Brazilian National Board for Ethics in Research (222/2006; 25000.001992/2006-17). Of note, only two individuals declined to participate and were not included in the study.

Given that this population-based study involved the recruitment of the entire Prata village, no inferential statistics were applied. Descriptive statistics was used to describe and compare two sub-populations: individuals born versus individuals not born in the village, the rationale being that, whereas no information about life conditions pre-Prata was available for the sub-population of individuals not born in the village, individuals born in the village have been living their entire lives in a homogenous environment, hyperendemic for leprosy. Median and mean age at diagnosis were estimated and depicted in a cumulative age plot.

## Results

A complete characterization of the population of the Prata village is summarized in Table 1. Of the 2,005 individuals enrolled, 1,672 self-reported as non-affected by leprosy, 319 self-reported as affected, and 14 did not disclose their leprosy status. Data verification confirmed 257 out of the 319 self-reported leprosy cases. For subsequent analyses, 76 individuals with unknown leprosy status (comprised of the 14 individuals who did not disclose their status and an additional 62 individuals with unavailable medical reports) were excluded. Consequently, the accumulated prevalence rate of leprosy for the period (including active and historical cases) was calculated as 12.8% (257 cases out of 2,005 individuals), evenly distributed across males and females. When stratifying the affected population by clinical form, 104 (40.5%) cases were lepromatous, 53 (20.6%) were tuberculoid, 53 (20.6%) were borderline, and 47 (18.3%) were indeterminate (Table 2). Therefore, the highest proportion of the Prata patients had a higher bacillary load.

**Fig. 1:**
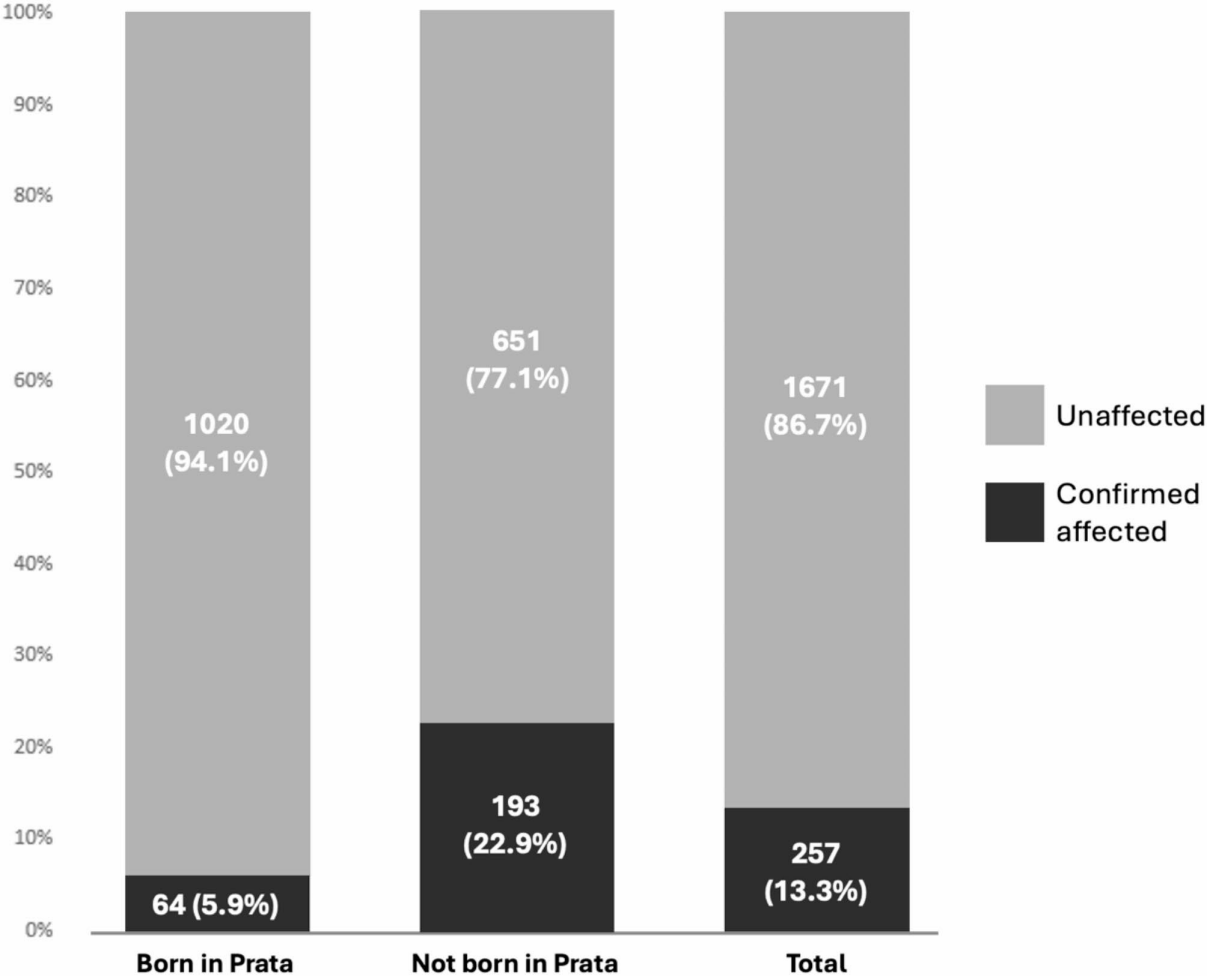
The proportion of affected individuals in the total population and the sub-populations of individuals born and not born at the Prata village. Numbers inside the bars: total number of individuals and proportion in %.

About half of the Prata village population—1,084 individuals (56.2%)—were born there. Among these, 64 were confirmed leprosy cases, resulting in a cumulative prevalence of 5.9%. Among the individuals not born in the village, the cumulative leprosy prevalence was 22.9% (193 confirmed cases/844 individuals) (Fig. 1). The median age at diagnosis among the individuals born in the village was 15 years old (mean = 18.1, ranging from 5 to 75 years old, standard deviation of ± 12.9). In contrast, the median age at diagnosis was 28 years old among the individuals not born in the village (mean = 30.1, ranging from 2 to 74 years old and standard deviation of ± 15.2) (Table 2, supplementary Fig. 1). A comparative description of the subpopulations born and not born in the village is presented in the supplementary Table 1.

**Table 1:** Epidemiological characterization of the confirmed affected and unaffected subjects in Prata Village.

	Confirmed affected subjects		Unaffected subjects		Total subjects	
	n	%	n	%	n	%
Total sample size (n)	257		1672		1929	
Age (years)						
n^a^	257		1668		1925	
Mean	47.8		20.3		24.0	
Median	49		16		19	
Minimum	10		0		0	
Maximum	89		82		89	
Standard deviation	19.9		15.9		19.0	
Sex						
Male	153	59.5%	819	49.0%	972	50.4%
Female	104	40.5%	853	51.0%	957	49.6%
Ethnicity						
White	27	10.5%	171	10.2%	198	10.3%
Black	50	19.5%	195	11.7%	245	12.7%
Mixed race	180	70.0%	1301	77.8%	1481	76.8%
Others	0	0.0%	4	0.2%	4	0.2%
Unknown	0	0.0%	1	0.1%	1	0.1%
Marital status						
Single	84	32.7%	1147	68.6%	1231	63.8%
Stable Union/Married	139	54.1%	474	28.3%	613	31.8%
Divorced	2	0.8%	9	0.5%	11	0.6%
Widowed	31	12.0%	38	2.3%	69	3.5%
Others	1	0.4%	3	0.2%	4	0.2%
Unknown	0	0.0%	1	0.1%	1	0.1%
Birthplace						
Prata	64	24.9%	1020	61.0%	1084	56.2%
Others	193	75.1%	651	38.9%	844	43.7%
Unknown	0	0.0%	1	0.1%	1	0.1%
Education						
Illiterate	66	25.7%	86	5.1%	152	7.9%
Incomplete Elementary School	92	35.8%	590	35.3%	682	35.3%
Complete Elementary School	19	7.4%	71	4.2%	90	4.7%
Incomplete Middle School	39	15.2%	312	18.7%	351	18.2%
Complete Middle School	7	2.7%	71	4.2%	78	4.0%
Incomplete High School	13	5.0%	110	6.6%	123	6.4%
Complete High School	11	4.3%	111	6.6%	122	6.3%
More than High School	3	1.2%	276	16.6%	279	14.5%
Unknown	7	2.7%	45	2.7%	52	2.7%
Alcohol drinking habit						
Yes	89	34.6%	337	20.2%	426	22.1%
No	168	65.4%	1335	79.8%	1503	77.9%
Smoking					0	
Yes	92	35.8%	292	17.5%	384	19.9%
No	165	64.2%	1380	82.5%	1545	80.1%
Recreative drug using habit						
Yes	5	1.9%	25	1.5%	30	1.5%
No	249	96.9%	1635	97.8%	1884	97.7%
Unknown	3	1.2%	12	0.7%	15	0.8%

n, number; n ^a^, number of individuals with available information.

**Table 2:** Clinical characterization of the Prata village—confirmed affected subjects.

	Confirmed affected subjects
Total sample size (n)	257
Age at diagnosis (years)	
n^a^	257
Mean	27.2
Median	25
Minimum	2
Maximum	75
Standard deviation	15.5
Age at diagnosis—born in Prata (years)	
n^a^	64
Mean	18.1
Median	15
Minimum	5
Maximum	75
Standard deviation	12.9
Age at diagnosis—not born in Prata (years)	
n^a^	193
Mean	30.1
Median	28
Minimum	2
Maximum	74
Standard deviation	15.2
Clinical classification	
Tuberculoid	53 (20.6%)
Borderline	53 (20.6%)
Lepromatous	104 (40.5%)
Indeterminate	47 (18.3%)

n, Number; n ^a^, number of individuals with the information available.

## Discussion

Here, we present a descriptive, population-based study of a well-controlled, hyperendemic, isolated leprosy-affected community that might bring insights into the epidemiology of this elusive disease. Our most striking findings are the difference between leprosy prevalence and age at diagnosis between two sub-populations of individuals born and not born in the village. We also describe a homogenous distribution of cases across males and females.

Leprosy cumulative prevalence among those born and those not born in the village was 5.9% and 22.9%, respectively. It is reasonable to assume that the high cumulative prevalence of leprosy among the population not born in the village is because many of these individuals moved to the village due to compulsory isolation, in place in Brazil at the time the village was designated as an isolation site (1923) and until the early-1960s. In contrast, the much lower cumulative prevalence among the subpopulation born in the village is aligned with previous estimates indicating that approximately 5% of the population is naturally susceptible to leprosy^7,8^, a proportion that seems to stand even in a hyperendemic, socially homogenous environment. Previous attempts to determine the proportion of individuals naturally susceptible to leprosy have yielded varied results, ranging from less than 1–12%^5,9^. It is important to highlight that susceptibility to leprosy depends on multiple factors, including genetics.

Three factors may contribute to the age-onset difference between the village subpopulations born and not born. First, more frequent epidemiological surveys in a village well known for being hyperendemic may lead to earlier detection and diagnosis; second, individuals born in the village are mostly descendants of leprosy-affected individuals and, therefore, likely more genetically susceptible to the disease^6^; finally, individuals born in the village may be under lifelong exposure to leprosy cases, potentially increasing their risk of infection. If true, the 15-year average age of diagnosis in this sub-population may indicate the time between early exposure and clinical manifestation of the disease. The occurrence of leprosy cases among children is considered an indicator of ongoing transmission in the community^10,11^, which supports our findings. The leprosy incubation period has been a research subject for several decades. It has been proposed that the disease has an average incubation period of approximately five years^4,12^. An early attempt to estimate the incubation period of leprosy based on patient self-reports reached 8.4 years^13^. In one study, data from secondary cases of leprosy among household contacts revealed an average time of disease onset of 4.3 (± 3.3) years, irrespective of the clinical form^14^; in this study, the time between the diagnosis of an index leprosy patient and the occurrence of a leprosy secondary case in a household contact was considered a proxy for incubation. It should be noted, however, that the study was performed in a hyper-endemic region; thus, the estimate may be biased by the assumption that exposure occurs only through the index case living in the same household. A study involving 35 military veterans reported a median onset time ranging from 2 to 5 years for tuberculoid and 8 to 12 years for lepromatous leprosy; however, these findings did not reach statistical significance^15^. Furthermore, estimates of a 15 to 30-year incubation period in animal models primarily rely on non-human primates, a non-consensus model for leprosy studies^15–17^. A screening of the available literature shows that many references of the incubation period refer to a book Chap^18^, describing it as being between 2 and 5 years; careful analysis of the references used in the book revealed that the information is based on studies of different experimental designs, resulting in findings ranging from 2 to 30 years. More recently, a case report was published about a male individual with an incubation period of 50 years before symptom onset^19^. Together, these studies emphasize the variability in the data related to the incubation period of leprosy.

Another interesting observation is the distribution of leprosy across sexes in the Prata population. While it is commonly reported that leprosy is more prevalent in males compared to females^20,21^, we found that this difference is not clear among sub-populations born and not born in the Prata Village. The proportions of males and females among both sub-populations were similar, with 51.5% males and 48.5% females among those born in the village and 48.9% males and 51.1% females among those not born in the village. Given that the disease is incorporated into the culture of the village and is thus more naturally perceived by the population, our data suggests that the sex effect on leprosy distribution may be more influenced by behavioral factors rather than inherent biological differences between males and females. This is supported by the lack of gender-related differences in prevalence observed among wild armadillos^22^ and wild chimpanzees^23^.

Importantly, if the population in the Prata Village indeed has increased natural susceptibility to leprosy, we would expect to observe a higher incidence of reinfection/relapse cases. This is supported by a recent study that reported high rates of leprosy recurrence in the village, along with a high prevalence of drug-resistant strains of *M. leprae*^24^. These observations suggest that the Prata Village population may be both (i) more susceptible to leprosy as well as (ii) exposed to resistant bacteria; the combination of these independent aspects of the disease pathogenesis creates an ideal environment for the persistence of leprosy hyperendemicity.

This study has several limitations to consider. For example, as we are confident in our strategy of cross-checking clinical records of different sources to characterize a case, we are also aware that clinical examination would be interesting, particularly for the self-reported non-affected population. However, examining the entire population of the village would be an enormous task; also, we believe that the risk of a self-reported non-affected individual being affected is reduced by (i) the fact that the village is maintained under constant surveillance, with an MD dermatologist visiting the village on regular basis, and (ii) because leprosy is widespread and part of the local culture, the stigma associated with the status of affected is reduced amongst the villagers, favoring seeking for medical help. Our observations also suggest that some villagers may perceive being diagnosed with leprosy as a positive outcome, as they may become eligible for financial governmental support^25^.

Another limitation, particularly regarding our arguments on the proportion of naturally susceptible individuals and the disease’s incubation period, is that one must assume equal and permanent exposure of all newborn individuals to *M. leprae* throughout their lives, which is impossible to prove. For example, our approach does not consider the exact clinical form of each leprosy case and the presence or absence of an index case within the household of a new case^26^. On the other hand, the Prata village presents (i) a high prevalence of leprosy (12.8%), (ii) most of the cases of high bacillary load (40.5% were lepromatous), and (iii) a homogenous environment with a low degree of social isolation between leprosy affected and non-affected individuals. These observations favor the assumption that the Prata population is under constant high exposure.

In summary, the opportunity to compare two distinct subpopulation samples sharing a geographically limited, homogenous environment revealed differences in both the cumulative prevalence and the median age of diagnosis of leprosy between the groups born versus not-born in the village. Interestingly, no sex-related differences were observed between the two groups. In conclusion, although extrapolating our findings to more open populations must be done carefully, we believe we used a unique population as a model to provide additional insights into the epidemiology of leprosy.

## Electronic supplementary material

Below is the link to the electronic supplementary material.

Supplementary Material 1

## Data Availability

The authors state that all data produced in the study is included in the manuscript. Inquires can be addressed to the corresponding author, Prof. Marcelo Távora Mira.
