# Supplementary material for: Insights into leprosy epidemiology from an isolated population located in the Brazilian Amazon
Source: Sci Rep. 2025 Feb 19;15:6103. doi: 10.1038/s41598-025-90399-0 (PMC11840054; doi:10.1038/s41598-025-90399-0)
Supplement: Supplementary file 1 — Supplementary Material 1 [file 41598_2025_90399_MOESM1_ESM.docx]

Supplementary Material

Leprosy epidemiology: insights from the Prata Village

Ciane Cristina de Oliveira Mackert^1▼^, Fernando Panissa Lázaro^1^, Márcia Olandowski^1^, Helena Regina Salomé D’Espindula^1,5^, Andressa Mayra dos Santos^1^, Priscila Verchai Uaska Sartori^1^, Rafael Saraiva de Andrade Rodrigues¹, Geison Cambri¹, Marília Brasil Xavier^2^, Erwin Schurr^3^, Alexandre Alcaïs^4^, Marcelo Távora Mira^1^*

^1^ Graduate Program in Health Sciences, School of Medicine and Life Sciences, Pontificia Universidade Católica do Paraná, Curitiba, Paraná, Brazil;

^2^ Core for Tropical Medicine, Federal University of Pará, Belém, Pará, Brazil;

^3^ Infectious Diseases and Immunity in Global Health, McGill University Health Centre, Montreal, Canada;

^4^ Laboratory of Human Genetics of Infectious Diseases, Institut National de la Santé et de la Recherche Medicale and University Paris René Descartes, Necker Medical School, Paris, France.

^5^ Department of Clinical Analysis, Universidade Federal do Paraná, Paraná, Brazil.

*^▼^ In memorian*

**Correspondence:***m.mira@pucpr.br

**Supplementary table 1**. Epidemiological characterization by the birthplace of the subjects in the study

|  | **Born in Prata** | | **Not born in Prata** | |
| --- | --- | --- | --- | --- |
|  | **n** | **%** | **n** | **%** |
| **Total Sample Size (n)** | 1084 |  | 844 |  |
| **Status** |  |  |  |  |
| Confirmed Affected Subjects | 64 | 5.9% | 193 | 22.9% |
| Unaffected Subjects | 1020 | 94.1% | 651 | 77.1% |
| **Age (years)** |  |  |  |  |
| n ^a^ | 1084 |  | 840 |  |
| Mean | 13.5 |  | 37.6 |  |
| Median | 12 |  | 36 |  |
| Maximum | 0 |  | 2 |  |
| Minimum | 80 |  | 89 |  |
| Standard deviation | 10.4 |  | 19.0 |  |
| **Sex** |  |  |  |  |
| Male | 558 | 51.5% | 413 | 48.9% |
| Female | 526 | 48.5% | 431 | 51.1% |
| **Ethnicity** |  |  |  |  |
| White | 110 | 10.1% | 88 | 10.4% |
| Black | 119 | 11.0% | 126 | 14.9% |
| Mixed Race | 854 | 78.8% | 626 | 74.2% |
| Others | 0 | 0.0% | 4 | 0.5% |
| Unknown | 1 | 0.1% | 0 | 0.0% |
| **Marital Status** |  |  |  |  |
| Stable Union / Married | 146 | 13.5% | 466 | 55.2% |
| Single | 936 | 86.3% | 295 | 34.9% |
| Divorced | 2 | 0.2% | 9 | 1.1% |
| Widowed | 0 | 0.0% | 69 | 8.2% |
| Others | 0 | 0.0% | 4 | 0.5% |
| Unknown | 0 | 0.0% | 1 | 0.1% |
| **Education** |  |  |  |  |
| Illiterate | 14 | 1.3% | 138 | 16.3% |
| Incomplete Elementary School | 375 | 34.6% | 307 | 36.4% |
| Complete Elementary School | 38 | 3.6% | 52 | 6.2% |
| Incomplete Middle School | 213 | 19.6% | 138 | 16.3% |
| Complete Middle School | 35 | 3.2% | 43 | 5.1% |
| Incomplete High School | 65 | 6.0% | 58 | 6.9% |
| Complete High School | 59 | 5.4% | 63 | 7.5% |
| More than High School | 253 | 23.3% | 26 | 3.1% |
| Unknown | 32 | 3.0% | 19 | 2.2% |
| **Alcohol drink** |  |  |  |  |
| Yes | 148 | 13.7% | 278 | 32.9% |
| No | 936 | 86.3% | 566 | 67.1% |
| **Smoking** |  |  |  |  |
| Yes | 112 | 10.3% | 272 | 32.2% |
| No | 972 | 89.7% | 572 | 67.8% |
| **Recreative Drug User** |  |  |  |  |
| Yes | 12 | 1.1% | 18 | 2.1% |
| No | 1067 | 98.4% | 816 | 96.7% |
| Unknown | 5 | 0.5% | 10 | 1.2% |
| **Treatment** |  |  |  |  |
| MDT | 55 | 85.9% | 133 | 68.9% |
| Unknown | 9 | 14.1% | 60 | 31.1% |

**Abbreviations:** n - Number; n ^a^ - Number of individuals who have the information available; MDT - Multidrug therapy.


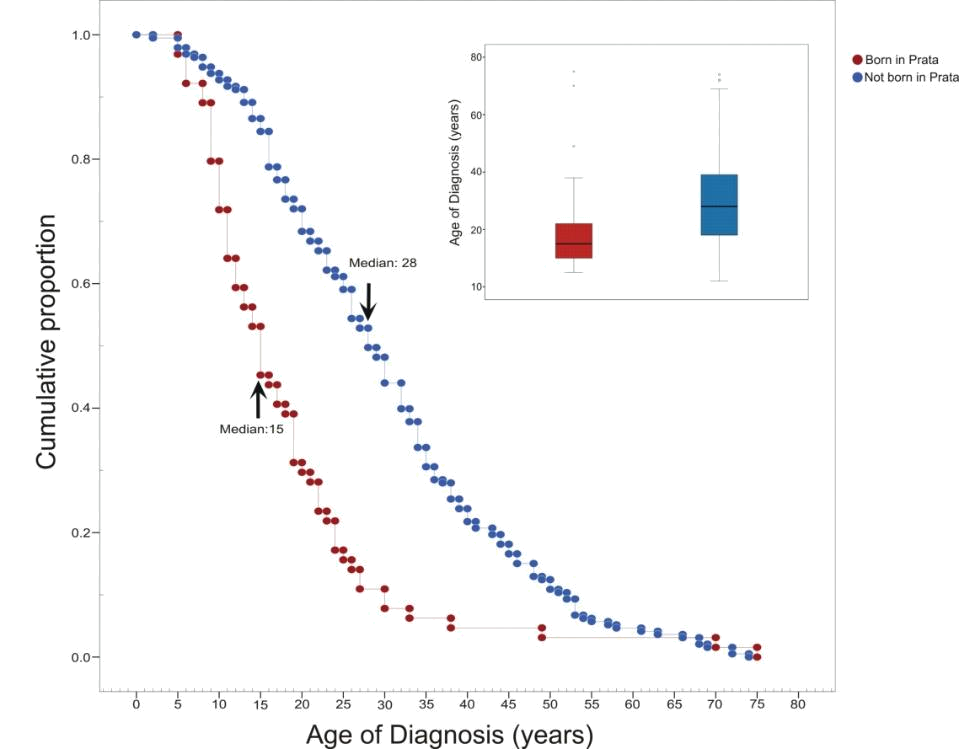


**Supplementary figure 1.** Cumulative age plot for age at diagnosis among affected individuals of the Prata Village, according to the place of birth (born vs. not born at the village). Not born in Prata (blue): sub-population of individuals that have not been born at the Prata village; Born in Prata (red): sub-population of individuals that have been born at the Prata Village. Kaplan-Meyer plot: Y-axis: the cumulative proportion of non-affected individuals; Median: median age at diagnosis. Box plot: the back bands inside the boxes are the median; the bottom and top of the boxes represent the first and third quartiles respectively; the ends of the whiskers represent the 10th and the 90th percentile.
